# Humanized Mouse Models of Epstein-Barr Virus Infection and Associated Diseases

**DOI:** 10.3390/pathogens2010153

**Published:** 2013-03-14

**Authors:** Shigeyoshi Fujiwara, Go Matsuda, Ken-Ichi Imadome

**Affiliations:** Department of Infectious Diseases, National Research Institute for Child Health and Development, Tokyo 157-8535, Japan; E-Mails: fujiwara-s@ncchd.go.jp (S.F.); matsuda-g@ncchd.go.jp (G.M.); imadome-k@ncchd.go.jp (K.I.)

**Keywords:** Epstein-Barr virus, humanized mouse, lymphoproliferative disease, EBV-associated hemophagocytic lymphohistiocytosis, rheumatoid arthritis, chronic active EBV infection, latent infection, immune responses, xenograft model.

## Abstract

Epstein-Barr virus (EBV) is a ubiquitous herpesvirus infecting more than 90% of the adult population of the world. EBV is associated with a variety of diseases including infectious mononucleosis, lymphoproliferative diseases, malignancies such as Burkitt lymphoma and nasopharyngeal carcinoma, and autoimmune diseases including rheumatoid arthritis (RA). EBV in nature infects only humans, but in an experimental setting, a limited species of new-world monkeys can be infected with the virus. Small animal models, suitable for evaluation of novel therapeutics and vaccines, have not been available. Humanized mice, defined here as mice harboring functioning human immune system components, are easily infected with EBV that targets cells of the hematoimmune system. Furthermore, humanized mice can mount both cellular and humoral immune responses to EBV. Thus, many aspects of human EBV infection, including associated diseases (e.g., lymphoproliferative disease, hemophagocytic lymphohistiocytosis and erosive arthritis resembling RA), latent infection, and T-cell-mediated and humoral immune responses have been successfully reproduced in humanized mice. Here we summarize recent achievements in the field of humanized mouse models of EBV infection and show how they have been utilized to analyze EBV pathogenesis and normal and aberrant human immune responses to the virus.

## 1. Humanized Mice in Virus Research

Mice have played a central role in biomedical researches including those on infectious diseases, as an easily accessible and versatile animal model. Abundant knowledge of their genetics promoted their use as a model system. Many human viruses, however, do not infect mice. Although murine viruses such as the mouse herpesvirus 68 (MHV-68) that is related to Epstein-Barr virus (EBV) and Kaposi’s sarcoma-associated herpesvirus (KSHV), and murine cytomegalovirus (CMV) homologous to human CMV have been considered as models of human viruses, there are considerable diversity in biological activity and pathogenesis between murine and human viruses [1,2,3]. Introduction of transgenes encoding virus receptors to mice enabled viruses such as adenoviruses [4], measles virus [5], coronavirus [6,7], and hepatitis C virus [8], to infect mice, but this approach did not remove the species barrier in many other viruses including human immunodeficiency virus 1 (HIV-1). A breakthrough in this issue was brought by the development of the *scid*-hu thy/liv mouse and the *scid*-hu PBL mouse in 1988 both of which are based on the C.B-17 *scid* mouse. Due to a mutation in the gene coding for a subunit of the DNA-dependent protein kinase that is essential for recombination of both the T-cell and B-cell receptor genes, C.B-17-*scid* mice lack both T and B cells and do not have the ability to reject human tissues and cells [9]. *Scid*-hu thy/liv mice were prepared by transplanting tissues of human fetal thymus and liver into the renal capsule of C.B-17 *scid* mice and allowed de novo generation of human T cells including the CD4^+^ compartment [10]. *Scid*-hu thy/liv mice were primarily used as an *In* *vivo* model of HIV-1 infection that targets mainly CD4^+^ T cells and monocytes/macrophages. In addition, *scid*-hu thy/liv mice were used for modeling of human herpesvirus 6 [11], human cytomegalovirus [12], Kaposi’s sarcoma-associated herpesvirus (KSHV) infections [13], measles virus [14], and varicella-zoster virus [15]. Various key features of human HIV-1 infection, including selective depletion of CD4^+^ T cells, were reproduced in *scid*-hu thy/liv mice [16,17,18]. *Scid*-hu PBL mice, on the other hand, were prepared by transplanting human peripheral blood mononuclear cells (PBMC) to the peritoneal cavity of C.B-17 *scid* mice and efficient engraftment of most components of the human immune system including T and B cells was observed [19]. *Scid*-hu PBL mice were used for modeling of HIV-1, human T-lymphotropic virus 1 (HTLV-1), and EBV infections and recapitulated many features of their pathogenesis [20,21,22,23]. There were, however, limitations in these early-generation humanized mouse models: human lymphocytes generated in *scid*-hu thy/liv mice did not distribute systemically and therefore intrathymic or intranodal HIV-1 inoculation was necessary and virus infection was mostly confined in the inoculated organs; *scid*-hu PBL mice were prone to graft *versus* host disease; both *scid*-hu thy/liv and *scid*-hu PBL mice did not mount primary immune responses to viruses. These disadvantages were overcome in new-generation humanized mice developed in this decade [24,25,26,27]. New-generation humanized mice were prepared by transplanting human hematopoietic stem cells (HSC) to mice of various severely immunodeficient strains (e.g., NOD/Shi-*scid* *Il2rg* ^null^ (NOG), Balb/c *Rag2*^−/−^*Il2rg*^−/−^ (BRG), and NOD/LtSz-*scid* *Il2rg*^−/−^ (NSG)) and major components of the human immune system, including T cells, B cells, NK cells, dendritic cells, and monocytes/macrophages, were reconstituted. In one protocol (BLT (bone marrow, liver, thymus) mouse), tissues of human fetal liver and thymus, as well as HSC derived from the same liver were transplanted and enabled proper intrathymic selection of human T cells [26]. These new-generation humanized mice can be infected with EBV [24,26,28,29,30,31], HIV-1 [32,33,34,35,36], HTLV-1 [37,38,39], dengue virus [40,41], herpes simplex virus type 2 (HSV-2) [42], and Kaposi’s sarcoma-associated herpesvirus (KSHV) [43]. In addition, humanized mice provided an opportunity to analyze human anti-influenza virus immune responses in mice, although infection with influenza virus *per* *se* had been feasible with regular mice [44,45]. Besides these immune system-humanized mice, immunodeficient mice harboring functioning human hepatocytes were developed and shown to be an excellent model for both hepatitis B and C virus infections and pathogenesis [46,47,48]. Furthermore, humanized mice harboring both human hepatocytes and human immune system components were prepared that could reproduce HCV-specific immune responses and pathogenesis [49]. Various immunodeficient mouse strains used in *In* *vivo* modeling of human virus infections are summarized in Table 1. In this review, we focus on humanized mouse models of EBV infection, especially new-generation humanized mouse models, and describe how they have been utilized for the reproduction of key features of human EBV infection, including pathogenesis, immune responses, and latent infection. Applications of these models, including preclinical studies on therapeutic strategies, are also discussed. 

**Table 1:** Humanized mouse strains used in virus research.

Strain of immuno- deficient mouse	Human cells reconstituted	Model of virus infection	Reference
Balb/c *Rag2*^−/−^*IL-2rg*^−/−^ (BRG) transplanted with HSC	T, B, dendritic cells	EBV, HIV-1, Dengue, HSV-2, influenza, HTLV-1	[24,29,33,41,42,45,50]
NOD/*scid* transplanted with human fetal liver, thymus and HSC (BLT)	T, B, NK cells, monocytes/ macrophages, dendritic cells	EBV, HIV-1,	[26,34]
NOD/Shi-*scid* *IL-2rg*^null^ (NOG) transplanted with HSC	T, B, NK cells, monocytes/ macrophages, dendritic cells	EBV, HIV-1, HTLV-1	[30,32,38,51,52,53,54]
NOD/LtSz-*scid* *IL-2rg*^−/−^ (NSG) transplanted with HSC	T, B, NK cells, monocytes/ macrophages, dendritic cells	HIV-1, EBV, CMV, Dengue, KSHV, adenovirus vector expressing HCV proteins	[31,55,56,57,58,59]
NOD/LtSz-*scid* *IL-2rg*^−/−^ (NSG) transplanted with human fetal liver, thymus and HSC (NSG-BLT)	T, B, NK cells, monocytes/ macrophages, dendritic cells	HIV-1, Dengue, EBV	[60,61,62,63]
*Rag2*^−/−^*IL-2rg*^−/−^ *Fah*^−/^^−^ transplanted with human hepatocytes	hepatocytes	HBV, HCV	[46]
*uPa*/*scid* mice transplanted with human hepatocytes	hepatocytes	HBV, HCV	[47,48]
Balb/c *Rag2*^−/−^*IL-2rg*^−/−^ (BRG) with a transgene encoding a fusion protein of the FK506 binding protein (FKBP) and caspase 8	hepatocytes, T cells, NK cells, plasmacytoid dendritic cells, myeloid dendritic cells	HCV	[49]

## 2. Overview on EBV

EBV is a ubiquitous lymphotropic γ herpesvirus infecting more than 90% of the adult population in the world [64]. Although primary EBV infection in childhood is mostly asymptomatic, that in adolescents and young adults results in infectious mononucleosis (IM) in at least 25% of cases [64]. Whether or not accompanied by IM, EBV infection results in life-long persistent infection. In a restricted fraction of hosts, EBV is etiologically involved in a variety of diseases, including lymphoproliferative diseases (LPDs) in immunocompromised patients such as posttransplant lymphoproliferative disease (PTLD) and AIDS-associated lymphoma, malignant lymphomas such as endemic Burkitt lymphoma and Hodgkin lymphoma, epithelial malignancies such as nasopharyngeal carcinoma and gastric carcinoma, and T/NK-cell LPDs such as chronic active EBV infection (CAEBV) and EBV-associated hemophagocytic lymphohistiocytosis (EBV-HLH). In addition, substantial evidence, although largely indirect at present, has accumulated implicating EBV in the pathogenesis of autoimmune diseases such as rheumatoid arthritis (RA) and multiple sclerosis. The list of most EBV-associated diseases is presented in Table 2. Some of these EBV-associated diseases show uneven geographical distribution: EBV-positive Burkitt lymphoma is endemic in equatorial Africa and New guinea; nasopharyngeal carcinoma is highly prevalent in southern China; EBV-associated T/NK LPDs are particularly prevalent in east Asia and natives in Central and South America; and parotic carcinoma is endemic in Alaskan Inuits. Obviously, etiological cofactors in addition to EBV, both environmental and host factors, are involved in the pathogenesis of these EBV-associated diseases, although majority of them are still unknown. EBV has a unique biological activity to transform human B lymphocytes into lymphoblastoid cell lines (LCLs) with the capacity of indefinite proliferation in culture. These EBV-transformed lymphoblasts are readily removed by the virus-specific cytotoxic T lymphocytes (CTL) in imunocompetent hosts [65,66]. In immunocompromised hosts like transplant recipients and AIDS patients, however, these transformed cells may proliferate unlimitedly to cause B-cell LPDs such as PTLD and AIDS-associated lymphoma. 

EBV genome encodes more than 80 genes and nine proteins (EBV nuclear antigens (EBNAs) 1, 2, 3A, 3B, 3C, and LP, and latent membrane proteins (LMPs) 1, 2A, and 2B) and two sets of untranslated RNAs (EBV-encoded small RNAs (EBERs) 1 and 2, and BamHI-A rightward transcripts (BARTs)) are expressed in B lymphoblastoid cells transformed by EBV. All or a part of these latent-cycle EBV proteins and RNAs are expressed in EBV-associated malignancies and LPDs. The pattern of EBV gene expression can be classified into three types: latency I, characterized by the expression of EBNA1, EBERs, and BARTs, is seen in Burkitt lymphoma and gastric carcinoma (some gastric carcinomas express LMP2 in addition); latency II, characterized by the expression of EBNA1, LMP1, LMP2A, EBERs, and BARTs, is found in CAEBV, Hodgkin lymphoma, and nasopharyngeal carcinoma; latency III, characterized by the expression of all nine EBV proteins and two sets of EBV RNAs expressed in LCLs, is seen in PTLD and AIDS-associated lymphoma [67].

EBV naturally infects only humans and has been reported to infect cotton-top tamarins, common marmosets, owl monkeys, and rabbits in experimental setting [68,69,70,71]. Cotton-top tamarins develop B-cell lymphomas following inoculation with EBV and the efficacy of candidate vaccines against the virus was evaluated in this model [72]. In spite of their potential value as an *In* *vivo* model of human EBV infection, they are endangered species and cannot be used in large numbers. A rhesus monkey lymphocryptovirus highly homologous to EBV was shown to reproduce key features of human EBV infection upon primary infection in naive monkeys, including oral transmission, atypical lymphocytosis, lymphadenopathy, expression of the B-cell activation marker in the peripheral blood, and sustained antibody responses to viral proteins [73]. This animal model has thus made significant contribution in the studies on EBV pathogenesis and treatment, although it has disadvantages common to primate models such as limited accessibility and high costs [74]. Intrinsic differences between the rhesus lymphocryptovirus and EBV needs also to be considered. Mouse models for EBV infection have not been available until the development of *scid*-hu PBL mice. Since the major target of EBV is lymphocytes, humanized mice are readily infected with the virus and EBV was actually the first human virus shown to infect new-generation humanized mice. 

**Table 2:** Epstein-Barr virus (EBV)-associated diseases.

Category	Diseases
Acute disease following primary infection	Infectious mononucleosis (IM)
Opportunistic infection	Posttransplant lymphoproliferative disease (PTLD), AIDS-associated lymphoma, oral hairy leukoplakia
T/NK-cell LPD^1^	Chronic active EBV infection (CAEBV), EBV-associated hemophagocytic lymphohistiocytosis (EBV-HLH)
Malignancies^2^	Burkitt lymphoma, Hodgkin lymphoma, Pyothorax-associated lymphoma, nasal NK/T-cell lymphoma, large granular cell leukemia, diffuse large B-cell lymphoma of the elderly, gastric carcinoma, nasopharyngeal carcinoma, thymic carcinoma, salivary gland carcinomas, leiomyosarcoma
Autoimmune diseases	Rheumatoid arthritis, multiple sclerosis, Sjögren syndrome, systemic lupus erythematosus

^1^Hydroa vacciniforme and hypersensitivity to mosquito bite are also included in this category. These two diseases may be manifest as an independent disease or as a complication to CAEBV. ^2^The rate of EBV positivity is different depending on the type of malignancy and also geographical location.

## 3. EBV Pathogenesis in Humanized Mice

### 3.1. Lymphoproliferative Disease

As described above, EBV induces B-cell LPD in immunocompromised patients and the most frequent histological type in PTLD and AIDS-associated lymphoma is diffuse large B-cell lymphoma (DLBCL), although smaller numbers of Burkitt lymphoma and Hodgkin lymphoma are also seen. Recapitulation of EBV-induced B-cell LPD in mice was first attained in *scid*-hu PBL mice. C.B-17 *scid* mice transplanted intraperitoneally with PBMC of EBV-seropositive donors developed acute B-cell lymphomas [19]. Subsequent analyses indicated that lymphoma cells were EBV-positive and expressed EBNA2 and LMP1, being consistent with the latency III program of EBV gene expression [75,76]. Histological examination and immunohistochemical staining of surface markers showed that this lymphoma in *scid*-hu PBL mice is similar to representative types of PTLD and AIDS-associated lymphoma [75,76]. Transplantation with PBL from EBV-seronegative donors, in contrast, did not induce lymphoma [19], but following inoculation with EBV a similar lymphoma with DLBCL histology was induced [21]. As expected, transplantation of established EBV-transformed LCLs also induced similar B-cell LPD in C.B-17 *scid* mice. Studies with *scid*-hu PBL mice provided valuable insights into the mechanism of EBV-induced lymphoproliferation *In* *vivo*. For example, engraftment of EBV-infected B cells in *scid*-hu PBL mice was found dependent on co-presence of uninfected CD4^+^ T cells, suggesting their essential role in *In* *vivo* proliferation and survival of EBV-transformed cells [77,78]. Involvement of human IL-10 and CXCL12/CXCR4 signaling in EBV-associated lymphomagenesis was elucidated in the *scid*-hu PBL mouse model [79,80]. Analysis on IFN-γ gene polymorphism indicated that the A/A genotype for the base +874 was more prevalent in PBLs inducing aggressive lymphoproliferation in *scid*-hu PBL mice. In biological analysis, the IFN-γ allele with adenosine at +874 was associated with inefficient CTL restimulation and this may explain the above finding [81].

These early humanized mouse models of B-cell LPD, however, had a number of limitations: first, no EBV-specific immune responses were detected in these mice and therefore investigation of immunosurveillance on EBV-associated LPD was not feasible; second, it was not possible to vary conditions of viral infection, including the dose and route of inoculation; third, analyses on the process of lymphomagenesis starting from viral inoculation to the development of lymphomas was not possible. Means to overcome these difficulties were provided when new-generation humanized mice were prepared by transplanting human HSC to immunodeficient mice of various strains [24,25,26,27]. Models of EBV infection based on new-generation humanized mice and features of human EBV infection reproduced in them are summarized in Table 3. Upon infection with EBV, these mice developed EBV-positive lymphoproliferation to various extents. Yajima and others characterized EBV-induced B-cell LPD in humanized NOG (hu-NOG) mice and determined various conditions for lymphomagenesis. They showed that EBV doses of more than 10^2^ 50% transforming dose (TD_50_) tended to cause LPD whereas those less than 10^1^ TD_50_ predominantly induced asymptomatic persistent infection. Expression of EBNA1, EBNA2, LMP1, and LMP2A was demonstrated in this B-cell LPD, indicating latency III type of EBV gene expression and immunohistochemistry showed the expression of CD23 and other B-cell activation markers. These findings, in combination with the observed DLBCL histology, indicated that EBV-associated LPD closely resembling PTLD and AIDS-associated lymphoma was reproduced in humanized mice [30]. Interestingly, Hodgkin-like cells with marked nucleoli and Reed-Sternberg-like cells with multiple nuclei, both of which are observed in primary EBV-positive DLBCL, were occasionally seen in this model of EBV-associated LPD. EBV-associated B-cell LPD with similar characteristics was also reproduced in humanized NSG (hu-NSG) mice and BLT NSG mice [31,62,63,82]. One of the earliest works on EBV infection of new-generation humanized mice used NOD/*scid* mice transplanted with human HSC where reconstitution of B cells and myeloid cells, but no T cells were demonstrated [28]. EBV-positive LPD generated in these mice is therefore considered to have developed without the influence of T-cell immunosurveillance. EBV gene expression of the latency II type was observed in this work [28]. 

EBV-associated LPD reproduced in humanized mice are an excellent target for preclinical studies of experimental therapies. A number of drug candidates and experimental therapies, including autologous EBV-specific CTL [83], IL-2-activated NK cells [84], anti-CTLA-4 antibody [85], rituximab and IL-2 in combination [86], GM-CSF and IL-2 in combination [87], low-dose IL-2 [88], and the combination of CD13/CD19-bispecific antibody, CD28 specific antibody, and autologous T cells [89], were evaluated using *scid*-hu PBL mice with EBV-positive LPD. One study using NOD/*scid* mice transplanted with PBMC with or without plasmacytoid dendritic cells depletion showed an important role for these cells in cellular immune responses against EBV-associated LPD [90]. EBNA2-driven expression of a thymidine kinase in EBV-transformed LCL cells transplanted to C.B-17 *scid* mice enabled complete regression of transplanted cells by the administration of ganciclovir [91]. Using NSG mice reconstituted with human HSC derived from fetal liver, Gurer and others demonstrated the effect of targeting EBNA1 to the DEC-205 endocytic receptor on dendritic cells. They prepared a fusion protein of EBNA1 carboxy terminus and the heavy chain of anti-DEC-205 antibody and showed that vaccination with this fusion protein primed EBNA1-specific IFN-γ secreting T cells and induced anti-EBNA1 antibodies [92].

### 3.2. EBV-HLH

Hemophagocytic lymphohistiocytosis (HLH) is a serious hyperinflammatory condition caused by a highly activated but ineffective immune responses [94]. Clinical features of HLH, mostly caused by overproduction of cytokines by CD8^+^ T cells and macrophages, include fever, hepatosplenomegaly, pancytopenia, hypertriglyceridemia, coagulopathy with hypofibrinogenemia, and liver dysfunction [94]. EBV-HLH is the most common and the severest type of virus-associated HLH and characterized by monoclonal or oligoclonal proliferation of EBV-infected T (most often CD8 ^+^ T) or NK cells [95,96]. Overproduction of cytokines by EBV-infected T cells as well as by activated macrophages and T cells reacting to the virus is thought to play a central role in the pathogenesis [97]. Sato and others reported on exaggerated CD8^+^ T-cell responses accompanied by IFN-γ cytokinemia in EBV-infected hu-NOG mice [53]. In contrast to earlier findings obtained from EBV-infected hu-NOG mice [30], they did not observe obvious EBV-positive LPD. EBV infection was restricted to B cells and expression of lytic-cycle genes was demonstrated. CD8^+^ T cells with activated phenotype infiltrated major organs including the liver and spleen and the mice exhibited normocytic anemia and thrombocytopenia. Based on these findings, together with the finding of hemophagocytosis in the bone marrow and other tissues, they suggested that EBV-infected hu-NOG mice might be useful as an experimental model for EBV-HLH. Although EBV-HLH is usually associated with EBV-induced proliferation of T or NK cells [95,96], their results suggest that EBV infection in B cells may also cause similar HLH. Indeed, in EBV-HLH that developed in patients with X-linked lymphoproliferative disease 1 (XLP-1) and XLP-2, proliferation of EBV-infected B cells was observed [98]. Using the same hu-NOG mice, Yajima and others observe predominantly B-cell LPD [30]. In fact, there were a number of differences in the protocols of Sato *et* *al.* and Yajima *et* *al.* and this may explain the differences in their results. For example, although both groups used cord blood-derived HSC, the former injected HSC to newborn male mice intrahepatically, whereas the latter used 6–8 week-old female mice and employed an intravenous route. The timing of virus infection after transplantation of HSC, an important factor determining the level of T-cell development [32], may also have an influence on the outcome of EBV infection of humanized mice. As described below, xenogeneic transplantation of PBMC obtained from patients with EBV-HLH to NOG mice also reproduced cardinal features of EBV-HLH [99]. 

**Table 3:** Humanized mouse models of EBV infection and associated diseases.

Mouse strain, age on transplantation, irradiation	Human cells and/or tissues transplanted, route of transplantation	Human immune system components reconstituted	EBV strain used, route of inoculation	Features of EBV infection reproduced	Reference
NOD/ *scid*, 8-10 weeks, 325 cGy γ irradiation,	CD34^+^ cells isolated from cord blood, intravenous	B cells, myeloid cells	Akata and EGFP- tagged B95-8, intrasplenic	B-cell LPD in latency II	[28]
Balb/c *Rag2*^−/−^*IL-2rg*^−/− ^(BRG), newborn, 4 Gy γ irradiation	CD34^+^ cells isolated from cord blood, intrahepatic	B, cells, T cells, dendritic cells	B95-8, intraperitoneal	B-cell proliferation, presumably EBV-specific T-cell response	[24,29]
NOD/ *scid*, 6-8 weeks, 325 cGy γ irradiation	Fetal thymus, fetal liver, liver-derived HSC (BLT mouse)	B cells, T cells, monocytes/macrophages, dendritic cells	Akata, intrasplenic	Human MHC-restricted T-cell response to EBV detected by ELISPOT assay	[26]
NOD/Shi- *scid* *IL-2rg*^null ^(NOG), 6-8 weeks, no irradiation	CD34^+^ cells isolated from cord blood, intravenous	B cells, T cells, NK cells, monocytes/macrophages, dendritic cells	Akata, itravenous, intraperitoneal	B-cell LPD, latent infection, erosive arthritis resembling RA, EBV-specific T-cell responses, IgM Ab to p18^BFRF3^	[30,51,52]
NOD/Shi- *scid* *IL-2rg*^null ^(NOG), newborn, 10cGy X irradiation	CD34^+^ cells isolated from cord blood, intrahepatic	B cells, T cells, NK cells, monocytes/macrophages, dendritic cells	Akata, intravenous	IFN-γ cytokinemia, hemophagocytosis, systemic infiltration of CD8^+^ T cells, signs of HLH	[53]
NOD/LtSz- *scid* *IL-2rg*^−/− ^(NSG), 2-5 days, 100 cGy irradiation	CD34^+^ cells isolated from fetal liver, intrahepatic	B cells, T cells, NK cells, monocytes/macrophages, dendritic cells	Unspecified, intraperitoneal	B-cell LPD, EBV-specific T-cell responses, establishment of EBV-specific T-cell clones	[31,82]
NOD/LtSz- *scid* *IL-2rg*^−/− ^(NSG) with HLA-A2 transgene, 2-5 days, 100-150 cGy irradiation	CD34^+^ cells isolated from fetal liver, intrahepatic	B cells, T cells, NK cells, monocytes/macrophages, dendritic cells	Unspecified intraperitoneal	EBV-specific T-cell responses restricted by HLA-A2	[31,93]
NOD/LtSz- *scid* *IL-2rg*^−/− ^(NSG), 6-10 week old, 2-3 Gy irradiation	Fetal thymus, fetal liver, liver-derived HSC (NSG-BLT mouse)	B cells, T cells, no description of other components	B95-8 recombinants (BZLF1 knocked-out or enhanced BZLF1 expression), intraperitoneal	B cell lymphoma with type I, type IIb, or type III latency, latent infection, EBV-specific T-cell responses	[62,63]

### 3.3. EBV-HLH

Hemophagocytic lymphohistiocytosis (HLH) is a serious hyperinflammatory condition caused by a highly activated but ineffective immune responses [94]. Clinical features of HLH, mostly caused by overproduction of cytokines by CD8^+^ T cells and macrophages, include fever, hepatosplenomegaly, pancytopenia, hypertriglyceridemia, coagulopathy with hypofibrinogenemia, and liver dysfunction [94]. EBV-HLH is the most common and the severest type of virus-associated HLH and characterized by monoclonal or oligoclonal proliferation of EBV-infected T (most often CD8 ^+^ T) or NK cells [95,96]. Overproduction of cytokines by EBV-infected T cells as well as by activated macrophages and T cells reacting to the virus is thought to play a central role in the pathogenesis [97]. Sato and others reported on exaggerated CD8^+^ T-cell responses accompanied by IFN-γ cytokinemia in EBV-infected hu-NOG mice [53]. In contrast to earlier findings obtained from EBV-infected hu-NOG mice [30], they did not observe obvious EBV-positive LPD. EBV infection was restricted to B cells and expression of lytic-cycle genes was demonstrated. CD8^+^ T cells with activated phenotype infiltrated major organs including the liver and spleen and the mice exhibited normocytic anemia and thrombocytopenia. Based on these findings, together with the finding of hemophagocytosis in the bone marrow and other tissues, they suggested that EBV-infected hu-NOG mice might be useful as an experimental model for EBV-HLH. Although EBV-HLH is usually associated with EBV-induced proliferation of T or NK cells [95,96], their results suggest that EBV infection in B cells may also cause similar HLH. Indeed, in EBV-HLH that developed in patients with X-linked lymphoproliferative disease 1 (XLP-1) and XLP-2, proliferation of EBV-infected B cells was observed [98]. Using the same hu-NOG mice, Yajima and others observe predominantly B-cell LPD [30]. In fact, there were a number of differences in the protocols of Sato *et* *al.* and Yajima *et* *al.* and this may explain the differences in their results. For example, although both groups used cord blood-derived HSC, the former injected HSC to newborn male mice intrahepatically, whereas the latter used 6–8 week-old female mice and employed an intravenous route. The timing of virus infection after transplantation of HSC, an important factor determining the level of T-cell development [32], may also have an influence on the outcome of EBV infection of humanized mice. As described below, xenogeneic transplantation of PBMC obtained from patients with EBV-HLH to NOG mice also reproduced cardinal features of EBV-HLH [99]. 

### 3.4. Rheumatoid Arthritis

Rheumatoid arthritis (RA), characterized by synovial proliferation and destruction of bone and cartilage tissues, is a common autoimmune disease associated with progressive disability and systemic complications [100]. Since the late 1970s, evidence has accumulated suggesting possible involvement of EBV in the pathogenesis of autoimmune diseases such as RA, systemic lupus erythematosus, Sjӧgren syndrome, and multiple sclerosis [101]. Evidence linking EBV and RA includes the following: in patients with RA, (i) circulating EBV load is higher than in healthy controls; (ii) activated CD8^+^ T cells specific to EBV are frequently detected; (iii) a large number of T cells specific to EBV-encoded proteins are present in affected joints; (iv) T-cell functions to suppress the outgrowth of EBV-induced B cells are deficient, and (v) larger numbers of EBV-infected B cells are present in the blood compared to normal controls [102]. Furthermore, infection with EBV and expression of EBERs and LMP1 were demonstrated in synovial cells of RA lesions [103]. These lines of evidence are, however, circumstantial and direct evidence for the causal relationship has been missing, primarily because of the lack of an appropriate animal model. Recently, Kuwana and others made histopathological analyses on joints of EBV-infected hu-NOG mice and recognized erosive arthritis with massive synovial cell proliferation and infiltration of human CD4^+^ T cells, CD8^+^ T cells, CD19^+^ B cells, and CD68^+^ macrophages [51]. Although few EBV-infected cells were found in the synovial membrane, a number of infected cells were demonstrated in the bone marrow adjacent to affected joints. Importantly, a histological structure termed pannus, very characteristic to RA and involved in the destruction of bone tissue was found in the arthritis lesions of hu-NOG mice. These results strongly suggest that EBV can trigger the development of erosive arthritis resembling RA in humanized mice. Since this study was limited to histopathological analyses, further studies on the pathogenesis of EBV-induced arthritis in humanized mouse models are necessary. Possible involvement of cytokines including TNF-α, IL-1, and IL-6, which are known to be involved in the pathogenesis of RA, can be readily tested by administration of specific antibodies and will indicate whether the arthritis in mice is generated by a similar mechanism as that in human RA. Because humoral immune responses to EBV was inefficient and only IgM antibody to a VCA component was detected in the virus-infected hu-NOG mice (see below), it is not likely that antigenic cross reactions between EBV proteins and synovial proteins play critical roles in the pathogenesis of arthritis. Indeed, rheumatoid factor and antibody to cyclic citrullinated peptides, two representative markers for RA, were not detected in mice presenting arthritis. If the mechanism of arthritis formation is shown to be common between humans and EBV-infected mice, preclinical studies on therapeutic strategies will be possible with humanized mice. 

## 4. EBV-Specific Immune Responses in Humanized Mice

Analysis on immune responses to EBV infection in humanized mice was made possible by the development of new-generation humanized mice [24,26]. Reconstitution of human B cells, T cells, and dendritic cells was achieved in BRG mice transplanted with human HSC as described above. Upon infection with EBV the number of T cells significantly increased in the spleen and CD8^+^ T cells isolated from infected mice were shown to proliferate vigorously when stimulated with autologous EBV-transformed LCL, although EBV specificity of this response was not confirmed [24]. In BLT mice developed by Melkus and others [26], the presence of human thymus tissue enabled restriction of T cells by human MHC and efficient EBV-specific T-cell responses was demonstrated. 

Subsequent to the pioneering works described above, EBV-specific T-cell responses have been studied in humanized mice of various strains [30,31,52,53]. EBV-specific T-cell clones of both CD4^+^ and CD8^+^ phenotypes were established from hu-NSG mice infected with the virus and specific lysis of HLA-matched LCL was demonstrated [31]. A few studies confirmed that antibodies specific to human class-I MHC inhibits production of IFN-γ by CD8^+^ T cells following stimulation with autologous LCL, indicating that the T cells isolated from infected mice recognize EBV epitopes presented by the human MHC class I [26,30]. Furthermore, human MHC class I tetramers presenting EBV epitopes identified EBV-specific CD8^+^ T cells in hu-NOG mice [53]. Since depletion of either CD4^+^ or CD8^+^ T cells by administration of anti-CD4 or anti-CD8 antibodies, respectively, caused more aggressive proliferation of EBV-infected cells and reduced the life span of infected mice, T-cell responses induced in humanized mice are thought to play a protective role [31,52]. One study further showed that CD8^+^ cells isolated from EBV-infected mice have the ability to suppress transformation of autologous B cells by EBV [52]. 

Induction of EBV-specific T-cell responses in BLT mice is readily acceptable because the presence of human thymus tissue is expected to allow proper selection of T cells by human MHC. However, T-cell responses specific to EBV have been demonstrated not only in BLT mice but also in other humanized mice without transplanted human thymus tissues [30,31,52,53]. Furthermore, T-cell responses specific to HIV-1 [104], an adenovirus vector expressing HCG glycoproteins [59], HSV-2 [42], and influenza virus [44] have been demonstrated in humanized mice without human thymic tissue. Since the positive selection of T cells in the thymus is thought to occur on the surface of epithelial cells and epithelial cells in humanized mice are of the murine origin, it is puzzling how T cells have developed with the capacity to recognize epitopes in the context of human MHC. Watanabe and others recognized a significant delay in the development of human T cells in humanized NOG I-A^−/−^ mice and this suggests that murine MHC is involved in the positive selection of human T cells [105]. Nevertheless, the fact that the development of human T cells was retained in humanized NOG I-A^−/−^ mice, although delayed and in a reduced number, suggests that human HSC-derived cells, possibly B cells or dendritic cells, are also involved in the positive selection of human T cells, explaining the T-cell responses restricted by human MHC in humanized mice [105]. One way to clear the problem of T-cell education is to introduce a human MHC transgene to humanized mice. Two NSG-derived mouse strains with human HLA-A2 transgene were developed and transplanted with HSC having the same HLA allele. Humanized mice thus obtained mounted efficient T-cell responses following EBV infection and restriction by HLA-A2 was demonstrated [31,93]. Although not tested in EBV infection so far, the *Rag1*^−/−^γc^−/−^ mouse with HLA-DR4 transgene were shown to have more human CD4^+^ T cells than control *Rag1*^−/−^γc^−/−^ mouse following reconstitution with HLA-DR4-positive HSC [106]. 

Antibody responses to EBV were analyzed in hu-NOG mice infected with EBV [30]. Compared with T-cell responses, antibody responses were much less effective; by examining 40 infected mice only three mice were found producing IgM antibody to P18^BFRF3^, a major component of the viral capsid antigen (VCA). No IgG antibody to EBV-encoded proteins were detected. Antibody responses to HIV-1 [32,33,104], dengue virus [41], and HSV-2 [42] have also been described in humanized mice. Although IgG response was reported for dengue virus and HSV-2 [41,42], most of these antibody responses have been restricted to IgM. This inefficiency in antibody response, especially that in IgG response, is probably not due to intrinsic defects in B cells in humanized mice because transfer of functional human T cells expressing TCR specific to hemagglutinin (HA) improved HA-specific IgG response in hu-NOG mice [105]. It was suggested that suboptimal interaction between T and B lymphocytes underlay the inefficient production of antigen-specific IgG antibodies in hu-NOG mice [105]. The lack of human follicular dendritic cells in humanized mice may be also involved in the inefficiency of class switching [24].

## 5. Persistent EBV Infection in Humanized Mice

EBV infection in immunocompetent hosts is mostly asymptomatic and EBV-infected lymphoblastoid cells with potential to unlimited proliferation are efficiently removed by the virus-specific CTL because they express highly immunogenic proteins such as EBNAs 3A, 3B, and 3C. In EBV latency in humans maintained by T-cell immunosurveillance, EBV reside in memory B cells where all viral protein expression is shut down, rendering them invisible to CTL. Persistent infection reminiscent of EBV latency in humans was reproduced in hu-NOG mice inoculated with low doses of the virus. Majority of hu-NOG mice inoculated with EBV of less than 10^1^ TD_50_ remained normal and survived for more than six months without apparent signs of diseases [30]. EBV DNA was detected in the peripheral blood only transiently for the several weeks following infection. When these mice were sacrificed more than six months post-infection, no macroscopic pathological changes were observed, yet a low number of EBER-positive cells were found in the tissues of spleen and lymph nodes. These cells were shown CD20-positive, but their morphology, with rather large cytoplasm, was not consistent with that of resting memory B cells [30]. RT-PCR analysis of RNA obtained from the spleen or liver of these persistently infected mice showed the expression of EBNA1, EBNA2, LMP1, and LMP2A, being consistent with the presence of latency III cells (Yajima *et* *al.* unpublished results). Thus, persistent EBV infection in hu-NOG mice did not appear to recapitulate all aspects of EBV latency in humans. Since a number of deficiencies have been observed in the functions of B cells in humanized mice [105], reproduction of *bone* *fide* EBV latency in memory B cells may require more sophisticated humanized mice. Nevertheless, it is an interesting question how immune responses are involved in the induction and maintenance of this persistent EBV infection in hu-NOG mice. Interestingly, EBV DNA level in the peripheral blood fluctuated in a few persistently infected mice and there the rise in EBV DNA level was immediately followed by the increase in CD8^+^ T cells and subsequent decline of EBV DNA level, suggesting an effective T-cell control of EBV-infected cells [52]. Trials to disrupt this persistent infection by immunosuppressive measures and induce EBV-associated LPD are underway. Cocco and others characterized EBV gene expression, surface marker expression, and hypermutation of immunoglobulin variable region in a single cell level in lymphoid tissues of humanized BRG mice infected with EBV [29]. They could identify EBV-infected cells of all three types of EBV latency (I, II, and III) in specific correlations with the location in the tissue and presence of hypermutation. Thus, the exact route by which EBV establishes latent infection in memory B cells, which is at present unclear, may be clarified in experiments using humanized mice. 

## 6. Characterization of EBV Mutants in Humanized Mice

Among the nine EBV proteins and the two sets of untranslated RNAs expressed in immortalized lymphoblastoid cells, EBNA2, EBNA3A, EBNA3C and LMP1 have been shown to play essential roles in the process of transformation, whereas knocking-out of the EBNA3B gene by homologous recombination did not affect the *in* *vitro* transforming ability of the virus [67]. Virus replication was not affected either. Nevertheless, as EBNA3B is well conserved in fresh clinical EBV isolates, a critical role for EBNA3B in the life cycle of EBV had been supposed. Recent work by White and others demonstrated that an EBV mutant with its EBNA3B gene knocked out induces more aggressive LPD in hu-NSG mice, suggesting that EBNA3B functions as a tumor suppressor gene [82]. B cells infected with this mutant virus secreted less T-cell chemoattractant CXCL10 and thereby escaped T-cell mediated killing. EBNA3B may thus help the host to control EBV-induced lymphoproliferation so that the virus should not give a life-threatening harm to the host. These findings were possible only in *In* *vivo* experiments with humanized mice and points to an important area for their application, namely *In* *vivo* characterization of virus mutants. 

BZLF1 is an immediate-early gene of EBV and acts as a switch from the latent to lytic cycle of EBV infection. Knocking-out of BZLF gene did not affect the virus’ ability to transform B cells *in* *vitro* and the involvement of BZLF1 in lymphomagenesis was not expected until experiments with humanized mice became feasible. Ma and others prepared an EBV recombinant with the BZLF1 gene knocked-out and that with enhanced BZLF1 expression and compared the efficiency of lymphoma genesis in BLT NSG mice [62,63]. The results clearly indicated that BZLF1 enhances lymphoma genesis by inducing abortive lytic infection. 

There are a number of EBV genes such as BHRF1 (encoding an Bcl-2-like anti-apoptotic protein) [107], BXLF1 (encoding EBV thymidine kinase) [108], BCRF1 (encoding viral IL-10) [109], loss-of-function mutants of which exhibited no or only minor phenotypic alteration in *in* *vitro* studies. Examination of these EBV mutants in humanized mice may reveal critical roles for these genes in EBV life cycle and pathogenesis. 

## 7. Mouse Xenograft Models of EBV-Associated T/NK-Cell LPD

Although B cells are the major target of EBV, in a group of diseases termed EBV-associated T/NK-cell LPDs, including CAEBV and EBV-HLH, the virus is mainly found in T or NK cells proliferating oligoclonally or monoclonally. CAEBV is characterized by prolonged IM-like symptoms, unusual patterns of antibody responses to EBV, and elevated EBV DNA load in the peripheral blood [110,111,112]. In the WHO classification of lymphomas [113], CAEBV corresponds largely with the systemic EBV^+^ T-cell lymphoproliferative diseases of childhood. Although monoclonal proliferation of EBV-infected cells implies malignant nature of the disease, chronic clinical time course and absence of morphological atypia in proliferating cells contradicts this notion, and the pathogenesis of this disease is largely unresolved. Overproduction of cytokines by EBV-infected T or NK cells and reacting T cells and macrophages is thought to be responsible for systemic inflammatory symptoms in CAEBV. Although it is still not possible to transform human T and NK cells *in* *vitro* with EBV to establish immortalized cell lines, the nature of these diseases strongly suggests that in a specific condition EBV can infect T and NK cells and induce their proliferation. EBV infection of T and NK cells has not been reproduced so far in humanized mice and recapitulation of EBV-associated T/NK LPD in mice required xenotransplantation of PBMC derived from patients. Imadome and others transplanted PBMC isolated from patients with CAEBV and EBV-HLH to NOG mice and succeeded in reproducing major features of these diseases including systemic monoclonal proliferation of EBV-infected T or NK cells and hypercytokinemia [99]. Many features were common to CAEBV and EBV-HLH model mice, but the findings of hemorrhagic lesions and extreme hypercytokinemia were unique to the latter model. Importantly, these models revealed an essential role of CD4^+^ T cells (whether or not infected with EBV) in the *In* *vivo* proliferation of EBV-infected T and NK cells and depletion of CD4^+^ T cells by administrating OKT-4 antibody just following transplantation of PBMC effectively prevented the engraftment of EBV-infected cells [99]. Furthermore, administration of OKT-4 antibody after engraftment of EBV-infected cells reduced peripheral blood EBV DNA load to undetectable level (Imadome and others, unpublished results). These results suggest therapeutic approaches targeting CD4^+^ T cells may be possible. 

## 8. Future Directions

### 8.1. Further Analyses on EBV Pathogenesis

EBV is implicated in a variety of diseases (Table 2) and only a minor fraction of them have been recapitulated in humanized mice. Efforts to reproduce the remaining diseases in humanized mice need to be made. Recognition of erosive arthritis resembling RA in humanized mice rationalizes a search for lesions and symptoms of other autoimmune diseases in EBV-infected humanized mice. By varying conditions for EBV infection in humanized mice, including viral dose, viral strain, route of inoculation, timing of infection after transplantation of HSC, as well as modifying the protocol for preparing humanized mice, recapitulation of additional EBV-associated diseases may be possible. As various environmental and host factors are thought to be involved in the pathogenesis of EBV-associated diseases, humanized mice may be a powerful tool for testing the effects of such cofactor candidates *in* *vivo*. For example, the effects of supposed cofactors for endemic Burkitt lymphoma such as malaria infection and euphorbia plants might be tested in humanized mice. Host genetic factors may be also evaluated in humanized mice; for diseases such as RA in which HLA polymorphism has an influence on pathogenesis, preparing humanized mice with HSC with high-risk polymorphisms may enhance pathogenesis. Similarly, primary immunodeficiency with specific susceptibility to EBV may be reproduced by preparing humanized mice with HSC derived from patients. 

### 8.2. Oral EBV Transmission

EBV is transmitted orally via saliva and initial steps of infection take place in oropharyngeal epithelium and lymphoid tissues just adjacent the epithelium. EBV inoculation to humanized mice so far, however, employed only intravenous or intraperitoneal routes and therefore critical early events in EBV infection may not have been reproduced there. Preliminary trials of oral inoculation of EBV to hu-NOG mice have not been successful (Yajima *et* *al.* unpublished result). Since no human epithelial cells are present in humanized mice, this result suggests that replication in epithelial cells is an essential step in primary EBV infection. Since oral transmission is a critical initial step in primary EBV infection that may direct later stages of EBV infection in the host, it is highly desirable that this natural route of transmission is reproduced in humanized mice. 

### 8.3. Innate Immune Responses to EBV

Human EBV infection is usually asymptomatic and the symptoms of IM appear only after long incubation period of 3–7 weeks. It is therefore extremely difficult to find individuals currently having acute primary EBV infection in a period suitable for analysis of innate immune responses. In this context, the humanized mouse may be an ideal tool and critical early innate responses to EBV might be revealed in humanized mice. Although not analyzing early events following infection, one study using NOD/*scid* mice with human fetal thymus xenograft focused on innate immune responses to EBV and demonstrated a role of EBV-induced CD8^+^ NKT cells in the suppression of tumorigenesis by EBV-associated Hodgkin lymphoma and nasopharyngeal carcinoma cells [114]. 

### 8.4. Improving Humanized Mice

Efforts to overcome various limitations in the current humanized mouse models are underway. For example, engraftment of human cells has been improved by introducing human SIRPα transgene to immunodeficient mouse strains or introducing murine CD47 gene to human HSC, thereby avoiding rejection by murine macrophages through improved SIRPα-CD47 signaling [115,116]. Because murine cytokines and growth factors are generally poorly cross-reactive with human receptors, supplementation of human equivalents either by direct injection, introduction of transgenes, or knock-in recombination is expected to improve reconstitution of human immune system components. Indeed, supplementation of human cytokines such as GM-CSF, IL-4, M-CSF, IL-7, IL-15, and EPO has been reported to improve the development and/or maintenance of certain human immune system components [117]. These improved protocols, as well as introduction of human MHC transgenes described above, will eventually realize humanized mice with the capacity of immune responses comparable to those in humans. Evaluation of vaccine candidates, including that for EBV, may become feasible with these improved humanized mice. 
